# 

**Published:** 1888-08-25

**Authors:** 


PRICE ONE PENNY.
No. 100, Vol. IV.—August 25th, 1888.
[Registered at the General Post Office as a Newspaper.]

Per Annum, 6s. 6d., post free.

Monthly Parts, 6d.; post free, 7½d.

Ditto per Ann., 7s. 6d. post free.

